# QTL Mapping in New *Arabidopsis thaliana* Advanced Intercross-Recombinant Inbred Lines

**DOI:** 10.1371/journal.pone.0004318

**Published:** 2009-02-02

**Authors:** Sureshkumar Balasubramanian, Christopher Schwartz, Anandita Singh, Norman Warthmann, Min Chul Kim, Julin N. Maloof, Olivier Loudet, Gabriel T. Trainer, Tsegaye Dabi, Justin O. Borevitz, Joanne Chory, Detlef Weigel

**Affiliations:** 1 Department of Molecular Biology, Max Planck Institute for Developmental Biology, Tübingen, Germany; 2 Plant Biology Laboratory, The Salk Institute for Biological Sciences, La Jolla, California, United States of America; 3 Section of Plant Biology, University of California Davis, Davis, California, United States of America; 4 Department of Ecology and Evolution, University of Chicago, Chicago, Illinois, United States of America; 5 INRA, Genetics and plant breeding - SGAP, Versailles, France; 6 School of Biological Sciences, The University of Queensland, St. Lucia, Australia; 7 Department of Biochemistry, University of Wisconsin, Madison, Wisconsin, United States of America; 8 Howard Hughes Medical Institute, The Salk Institute for Biological Sciences, La Jolla, California, United States of America; Purdue University, United States of America

## Abstract

**Background:**

Even when phenotypic differences are large between natural or domesticated strains, the underlying genetic basis is often complex, and causal genomic regions need to be identified by quantitative trait locus (QTL) mapping. Unfortunately, QTL positions typically have large confidence intervals, which can, for example, lead to one QTL being masked by another, when two closely linked loci are detected as a single QTL. One strategy to increase the power of precisely localizing small effect QTL, is the use of an intercross approach before inbreeding to produce Advanced Intercross RILs (AI-RILs).

**Methodology/Principal Findings:**

We present two new AI-RIL populations of *Arabidopsis thaliana* genotyped with an average intermarker distance of 600 kb. The advanced intercrossing design led to expansion of the genetic map in the two populations, which contain recombination events corresponding to 50 kb/cM in an F_2_ population. We used the AI-RILs to map QTL for light response and flowering time, and to identify segregation distortion in one of the AI-RIL populations due to a negative epistatic interaction between two genomic regions.

**Conclusions/Significance:**

The two new AI-RIL populations, EstC and KendC, derived from crosses of Columbia (Col) to Estland (Est-1) and Kendallville (Kend-L) provide an excellent resource for high precision QTL mapping. Moreover, because they have been genotyped with over 100 common markers, they are also excellent material for comparative QTL mapping.

## Introduction

Deciphering the genetic basis of natural variation in quantitative traits presents a challenge because the variation is often continuous and because there is often extensive genotype×environment (G×E) interactions. An effective way towards identifying the causal sequence variants includes identification and molecular characterization of quantitative trait loci (QTL). Despite substantial progress in cloning QTL genes, and even reducing some of them to Quantitative Trait Nucleotides (QTNs), QTL mapping and cloning remain a formidable task [1]–[8]. One of the major impediments is that QTL mapping typically produces large genetic intervals, which make it difficult to determine the best candidates for the causal genes. In addition, QTL of large effect can split into multiple QTL, with each explaining only a small proportion of the total variance.

An important factor influencing QTL confidence intervals is the number of recombination events in the mapping population. Therefore, if one increases the number of recombination events in each individual, precision improves without the need to phenotype a larger number of individuals. However, the success of this approach is dependent on how densely the population is genotyped, and requires a wealth of molecular polymorphism information across populations, which can then be exploited to develop markers for genotyping. Fortunately, in many species, mapping markers are no longer a rate-limiting factor. In the plant *Arabidopsis thaliana*, more than 300,000 non-singleton single nucleotide polymorphisms (SNPs) have recently been identified, for an average density of two to three SNPs per kb [9]. This has further improved the value of *A. thaliana* for ecological and evolutionary genetics [10], [11].

Recombinant Inbred Lines (RILs) are very useful in QTL analysis as they represent unique combinations of parental genotypes, and being immortal, they can be used for the analysis of many traits in many environments [12]. During the production of RILs, there are additional opportunities for recombination during the selfing generations, compared to simple F_2_ populations. A further improvement is the advanced intercross approach, in which beginning with the F_1_ or F_2_ generation, individuals are randomly intercrossed, thus increasing the opportunity of recombination before genotypes are fixed upon selfing [13].

Several *A. thaliana* RIL populations have been used for QTL mapping and subsequent molecular identification of the responsible genes [14]–[30]. However, there are relatively few large populations that have been densely genotyped, limiting the resolution of QTL maps (Table S1). Here we describe two RIL populations generated using an advanced intercross (AI) design, which captures an increased number of recombination events. The map resolution of the AI-RIL populations, which have been genotyped with over 180 markers, is roughly equivalent to what one would expect in 800 F_2_ individuals. We demonstrate the usefulness of the AI-RILs by mapping QTL for two traits, hypocotyl elongation and flowering time. In addition, we also identify two regions contributing to segregation distortion in one population.

## Results and Discussion

### RIL populations and genetic maps

**Figure 1:**
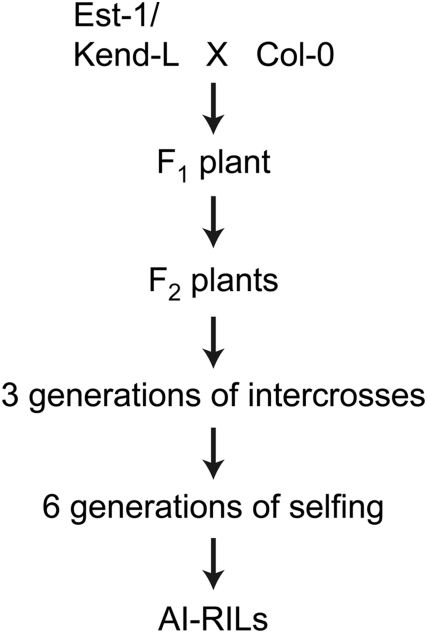
Diagram of the generation of AI-RILs. Intercrosses were between non-overlapping individuals.

The accessions Est-1 (Estland [Estonia]; CS6701) and Kend-L (Kendalville-Lehle; Lehle-WT-16-03) were crossed to the common lab strain Col (Columbia) as female. From the F_2_ progeny, 75 non-overlapping pairs of plants were intercrossed for three generations to create advanced intercross lines. The resulting lines were taken through six rounds of selfing without any intentional selection (Figure 1). The resulting 279 EstC (Est-1×Col) lines and 282 KendC (Kend-L×Col) lines were genotyped at 224 and 181 markers, respectively (Tables S2, S3). The markers were drawn from a recently published set of SNPs that distinguish the Col-0 reference strains from many other accessions [31], [32], as well as 45 SSLPs used for EstC (Table S2). There were 126 common markers for which Est-1 and Kend-L shared the same allele, which allows for combining mapping information across the two RIL populations. Genotyping was carried out using the MassARRAY platform [33].

**Figure 2:**
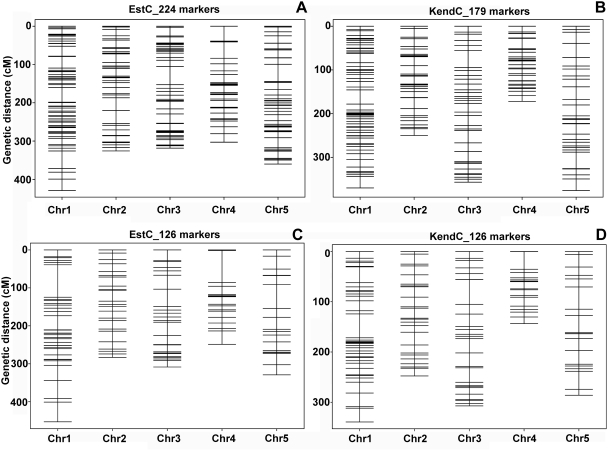
Genetic maps of AI-RIL populations. (A) Genetic map of EstC AI-RILs generated from the genotypes of 279 plants at 224 markers. (B) Genetic map of KendC AI-RILs generated using genotypes of 282 plants at 179 markers. (C, D) Genetic maps generated using the common 126 markers for the EstC (C) and KendC (D) populations. Note population-specific patterns of increases in genetic distances.

The genetic maps for both AI-RIL populations were similar, with an average of nearly two markers per Mb and no gap between markers greater than 30 cM. Due to the advanced intercross design, the genetic distances were increased between markers compared to regular RILs, resulting in an expanded map with higher resolution (Figures 2A, B). The expansion of the two populations differed to some extent, which is revealed when the maps are compared directly using the 126 common markers (Figure 2C, D). The variation potentially reflects differences in recombination rates between the populations.

### Segregation distortion

Many of the available *A. thaliana* RIL populations feature segregation distortion in selected regions of the genome [17], [20], [21], [26], [34]. Segregation distortion suggests either involuntary selection against these regions during generation of the RILs, or some type of incompatibility between genomic regions contributed by the different parents. For example, it has been reported that there are potential cases of reciprocal gene loss for paralogs located in segmental duplications, leading to some F_2_ combinations lacking either functional copy [35].

**Figure 3:**
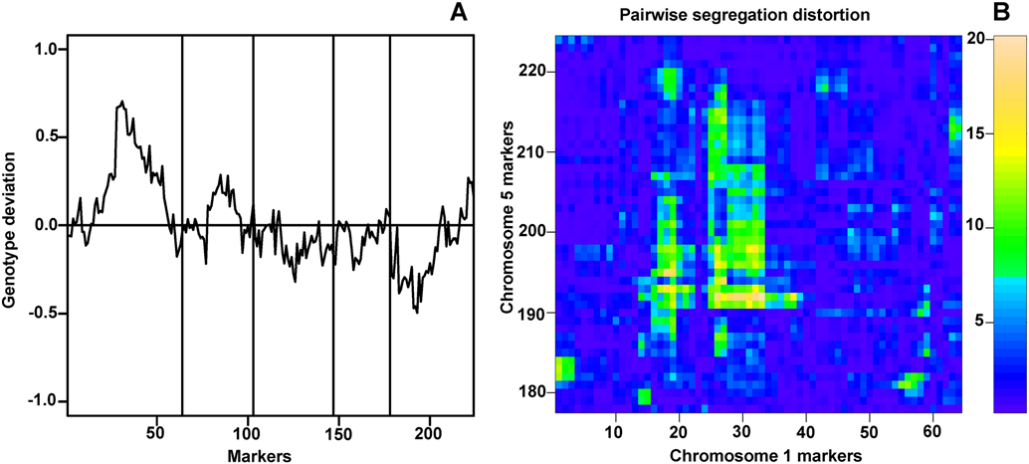
Segregation distortion in EstC AI-RILs. (A) Genotype of 224 markers over all five chromosomes. A value of 1.0 for a given marker represents all Est-1 alleles, while −1.0 corresponds to all Col alleles. (B) Chi-squared plot of pairwise segregation distortion for each marker on chromosome 1 and chromosome 5. Only the interaction between chromosome 1 and 5 markers are shown. Markers were consecutively numbered starting from the Northern most marker on chromosome 1.

While the KendC population did not show precise 50∶50 segregation for all markers, there were no regions with strong deviation from this expectation. In contrast, we observed extreme segregation distortion in the EstC population. On chromosome 1, between 14 and 16 Mb, the Col alleles were substantially underrepresented, with only about one third of the expected frequency. The converse was found around 8.4 Mb on chromosome 5, where only 25% of the population is homozygous for the Est-1 allele (Figure 3A; Table 1). A chi-squared test suggested that distortion for the two regions was not independent (Figure 3B). Indeed, not a single AI-RIL was simultaneously homozygous for Col alleles in the chromosome 1 region and for Est-1 alleles in the chromosome 5 region.

**Table 1:** Distribution of genotypes at markers on chromosomes 1 and 5 in the EstC AI-RIL population.

Chromosome	Position (Mb)	Est allele	Col allele	Total
1	14.2	219	38	257
1	15.2	225	44	269
1	15.4	224	43	267
1	15.9	226	46	272
1	16	218	45	263
5	8.3	71	196	267
5	8.4	68	201	269
5	8.6	72	194	266

To obtain additional information about the underlying cause of this interaction, we analyzed 500 F_2_ plants at the most biased SSLP marker in each region (Table 2). While two markers on chromosome 4 segregated as expected (1∶2∶1 for homozygous and heterozygous classes, with an overall distribution of 1∶1 for both alleles; data not shown), the alleles at chromosome 1 and 5 that were rare in the AI-RIL population were also rare in the F_2_ population. In addition, none of the F_2_ plants were Col homozygous at the chromosome 1 region and simultaneously homozygous for Est-1 at 8.4 Mb on chromosome 5. Therefore, the F_2_ analysis recapitulated the EstC population results, indicating that the Col region at chromosome 1 is incompatible with an Est-1 region at chromosome 5. The F_2_ experiment also demonstrates that the missing genotypes must be eliminated at or before the seedling stage, since DNA for genotyping was isolated from young adult plants. The same two genomic regions also show segregation distortion in a number of other *A. thaliana* RIL populations, and the causative loci are being pursued (O. Loudet, unpublished results).

**Table 2:** Chi-squared analysis of the genotypes in 422 F_2_ plants derived from a cross between Est-1 and Col.

Genotypes		Observed (O)	Expected (E)	(O-E)	(O-E)^2^	(O-E)^2^/E
Chr_1	Chr_5					
Col	Col	32	26.4	5.6	31.7	1.2
Col	Est	0	26.4	−26.4	695.4	26.4
Col	Het	36	51.7	−15.7	246.5	4.8
Het	Col	43	51.7	−8.7	75.7	1.5
Het	Est	15	51.7	−36.7	1346.9	26.1
Het	Het	130	105.5	24.5	600.3	5.7
Est	Col	48	26.4	21.6	467.9	17.7
Est	Est	28	26.4	1.6	2.7	0.1
Est	Het	90	51.7	38.3	1466.9	28.4
					**Chi-square sum**	111.76*

* p<0.0001

The mutually exclusive nature of the two regions makes epistatic analysis of loci involving these regions of the genomes problematic. Moreover, segregation distortion can affect marker order, as it creates spurious linkages. However, the availability of the genome sequence allowed us to fix the order of markers, which reduced the effects of segregation distortion on the genetic and QTL maps. Nevertheless, one needs to be aware that QTL located in these regions might not be properly identified.

### Hypocotyl length QTL

To evaluate the power of the AI-RIL populations we assayed two traits under complex genetic control, hypocotyl length and flowering time, both of which are influenced by the light environment. Since hypocotyls were measured in seedlings, while flowering time was determined in adult plants, the comparison between the two traits has the potential to provide information about light responsiveness at different developmental stages.

**Figure 4:**
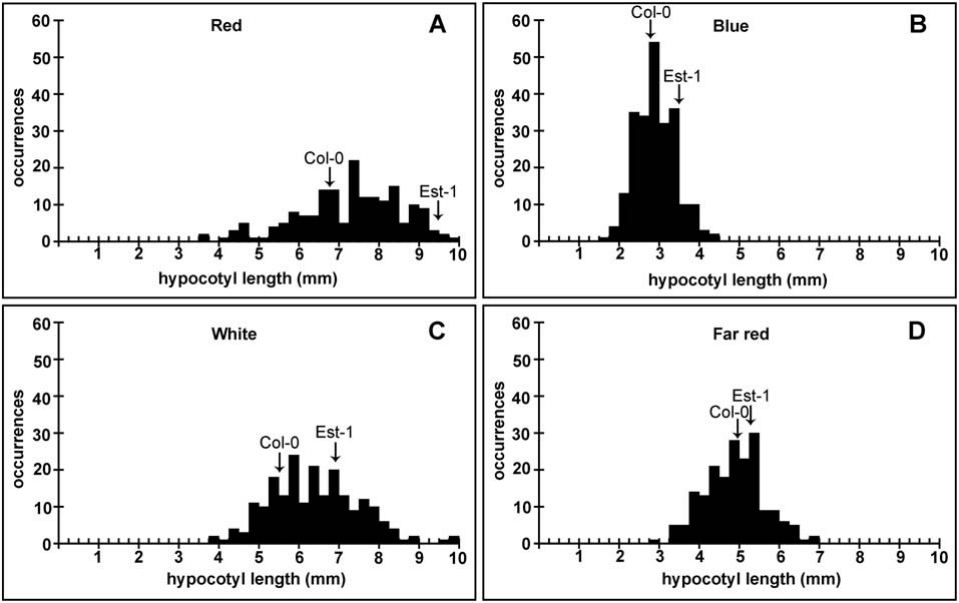
Variation in light response of EstC AI-RILs. Distributions of hypocotyl lengths under red (A), blue (B), white (C), and far-red (D) light are shown.

**Figure 5:**
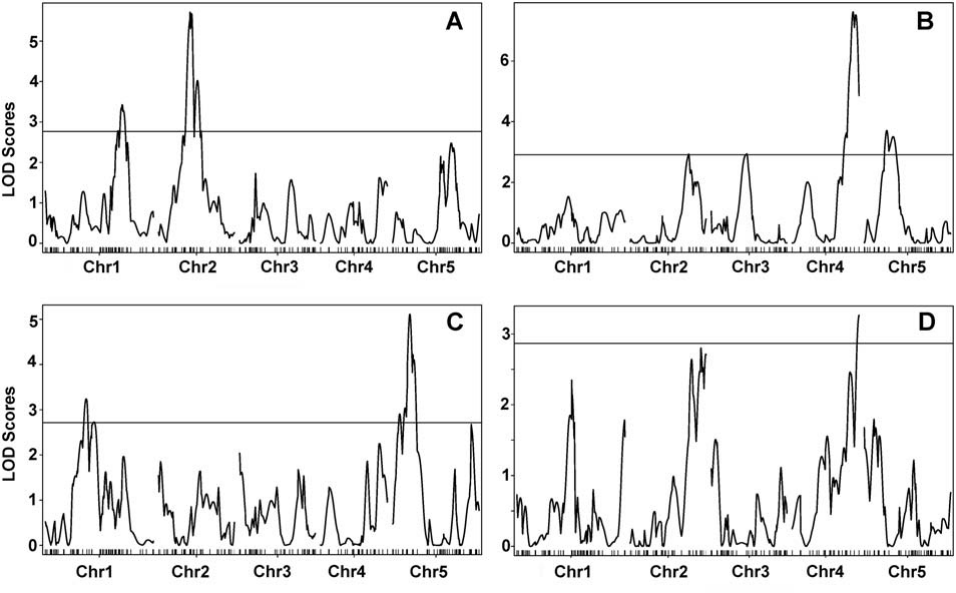
QTL analysis of hypocotyl elongation using EstC AI-RILs. QTL maps for red (A), blue (B), white (C), and far-red (D) are shown. Significant LOD scores were determined by permutation testing and are indicated by horizontal lines.

After seedlings germinate, they forage for light as an indicator of having broken the soil surface. Depending on the light environment, their embryonic stems, the hypocotyls, will be of different lengths. This trait is affected by both light quality and quantity, with seedlings grown in the dark having the longest hypocotyls. There is substantial variation in hypocotyl length among natural *A. thaliana* accessions, and several QTL for light-dependent hypocotyl length have been identified [36]–[38]. We analyzed the EstC population in four different light conditions (white, blue, red, far-red) and darkness. We found considerable variation in response to the four different light conditions along with transgression, suggesting that multiple loci contribute to variation in the EstC population (Figure 4; Table S2). Significant QTL, determined by permutation testing, were identified in all environments except darkness (Figure 5; Table S4). In white light, two QTL were significant, with a major-effect QTL detected on the top of chromosome 5 (Figure 5C), while in far-red light, a single marginally significant QTL was detected on the bottom of chromosome 4 (Figure 5D). In red light (Figure 5A), one QTL was identified on chromosome 1 (centered at 23.22 Mb), and a second one on chromosome 2 (centered at 8.20 Mb). The confidence interval for the second QTL in chromosome 2 included the *PHYB* locus (8.15 Mb). Est-1 has three polymorphisms compared to Col in the *PHYB* coding region, a 12 bp deletion near the initiation codon, and two nonsynonymous substitutions (I143L and L1072V). Among these, the I143L polymorphism has been recently to be associated with variation in red light response, making *PHYB* a good candidate for this QTL in the EstC population [39].

**Figure 6:**
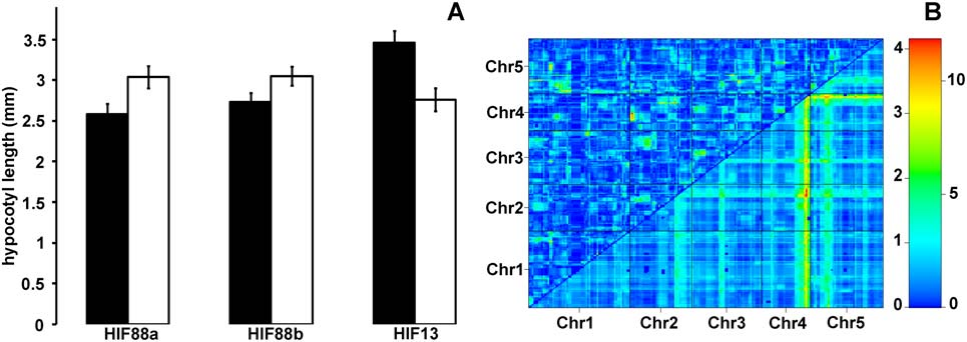
Confirmation of the chromosome 4 and 5 QTL for blue light response. (A) Comparison of hypocotyl lengths of heterogeneous inbred families segregating for the chromosome 4 (HIF88a and HIF88b) and 5 (HIF13) QTL. Col alleles are shown as black bars and Est-1 alleles as white bars. Error bars represent 95% confidence intervals. (B) A *scantwo* analysis of blue light hypocotyl lengths. Top triangle shows epistasis, bottom shows additive interactions. Color scale indicates LOD scores for epistasis (left) and additive interactions (right).

In blue light, one major QTL (*blue4*) was detected on the bottom of chromosome 4 (Figure 5B). We confirmed *blue4* both by repeating the phenotyping for a subset of AI-RILs (data not shown) and by exploiting heterogeneous inbred families (HIFs) derived from RIL 88, which is still heterozygous at the QTL (Figure 6A) [40]. QTL for hypocotyl length in this region, on the bottom of chromosome 4, have been previously identified in another RIL population in white, blue and red light conditions, suggesting that this region of the genome may harbor a QTL important for seedling light responsiveness [38]. However, given that the EstC QTL is detected only in blue light, it is possible that the causal gene in this case is different. Although several additional QTL peaks barely crossed the significance threshold on their own, a *scantwo* analysis (Figure 6B) revealed strong additive interactions for two regions (chromosomes 2 and 5) with the chromosome 4 QTL. Therefore, using HIFs derived from RIL 13, we tested and confirmed the interacting chromosome 5 QTL, demonstrating that this marginally significant QTL peak has significant phenotypic effects, and acts with *blue4* in an additive manner (Figure 6B).

### Flowering time QTL

**Figure 7:**
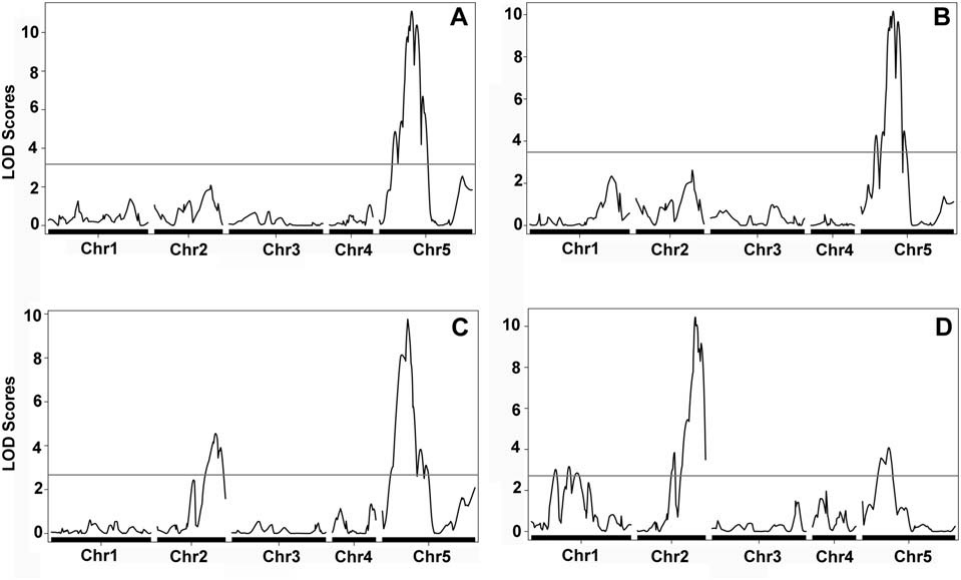
QTL analysis of flowering time in KendC AI-RILs. (A) QTL for days to flowering (DTF) in long days. (B) QTL for total leaf number (TLN) in long days. (C) QTL for DTF in short days. (D) QTL for TLN in short days. Note the magnitude of QTL effect changes with respect to the phenotype (DTF or TLN) in short days indicating a variation in growth.

Flowering time, which is thought to be important for reproductive success in the wild, has been found to be highly variable among natural *A. thaliana* accessions [26], [34], [41]–[43]. We analyzed flowering time in the KendC population in both long days, which promote rapid flowering in many *A. thaliana* strains, and in short days (Table S3). Flowering time was measured using days to flowering (DTF) as well as the total number of leaves (TLN), partitioned into rosette and cauline leaves. QTL analysis (Figure 7A, B) identified a single strong QTL for both DTF and TLN in long days on the top of chromosome 5, with the Kend-L allele delaying flowering. A single marker association analysis revealed that the Kend-L alleles in the QTL region accounted for six to seven additional leaves in long days. This QTL encompasses a region that contains several flowering time genes. Foremost among them is *FLOWERING LOCUS C (FLC),* a floral repressor that explains much of the natural variation in *A. thaliana* flowering [42]–[45]. Sequence analysis of the Kend-L allele of *FLC* revealed an insertion at the first intron of *FLC.* Intronic insertions at *FLC* typically lead to early flowering [42]–[45]. In this case, however, the Col allele confers early flowering, making *FLC* a less likely candidate for the QTL. Further fine mapping is needed to confirm the causative gene(s) for the QTL.

A strong QTL for DTF was detected on top of chromosome 5 in short days as well (Figure 7C). While its position is similar to that of the chromosome 5 QTL observed in long days (Figure 7A,B), the markers with the strongest association for either QTL are two Mb apart, suggesting that different genes underlie each of these QTL. In contrast, the use of TLN as a proxy for flowering time identified a strong QTL on the bottom of chromosome 2, which was not found in long days (Figure 7B, D). In addition, the two QTLs detected in short days (chromosomes 2 and 5) differed in their magnitude, with the chromosome 5 locus being the major QTL for DTF (Figure 7C), and the chromosome 2 locus being the major QTL for TLN (Figure 7D). This difference in QTL underlying traits that are genetically correlated [42] indicates variation in developmental growth rate under short day conditions.

### Conclusions

We have demonstrated the usefulness of the AI-RIL approach for *A. thaliana*. The two AI-RIL populations we describe, EstC and KendC, have been genotyped with well over a hundred markers, most of which are in common for both populations. Due to the large number of fixed recombination events, the size of the AI-RIL sets, and the density of markers, these populations provide an excellent resource for QTL analysis (Table S4).

## Materials and Methods

### Plant material

The RIL parents Est-1 (Estland; CS6701; male) and Kend-L (Kendalville, MI USA; Lehle-WT-16-03; male) were obtained from the ABRC (Arabidopsis Biological Resource Center, Ohio State University, OH, USA) and from Lehle Seeds (Round Rock, TX, USA), respectively. The final AI-RIL populations have been submitted to the *Arabidopsis* stock center and are available under stock numbers CS39389 (EstC) and CS39697 (KendC).

### Hypocotyl length analysis

Seeds were sterilized in 1.5-ml microcentrifuge tubes for 10 min in 70% ethanol, 0.01% Triton X-100, washed in 95% ethanol, and resuspended in sterile water. 60–70 seeds for each RIL were imbibed overnight and spotted individually on plates containing ½ Murashige and Skoog salts, 0.7% Phytagar with sufficient space to avoid seedling shading. Plates were placed at 4°C in the dark for 3 days, exposed to white light (120 μE m^−2^ sec^−1^) for 4 hours to induce germination (with the exception of plates destined for far-red conditions, were exposure to white light was increased to 12 hours to overcome any far-red induced inhibition of germination), and placed under the appropriate condition. Blue, red and far-red light treatments were conducted in Percival E30LED chambers (Percival Scientific, Boone, IA USA), while white light and dark treatments were carried out in a Percival E30B chamber with the dark sample wrapped in aluminum foil. Seeds were grown in white (40 μE m^−2^ sec^−1^), blue (4.2 μE m^−2^ sec^−1^), red (35 μE m^−2^ sec^−1^), or far-red (0.5 μE m^−2^ sec^−1^) light and the dark at 22°C for 7 days. Fluence rate was determined by using a Li-Cor1800 spectroradiometer (Li-Cor Biosciences, Lincoln, NE USA).

Analyses in all five conditions were done in the same week to minimize week-to-week variation of growth conditions and seed quality. Germination was recorded in white light conditions, in 12 hour intervals. The germination scores (0–3) were treated as a phenotype. QTL mapping with this phenotype did not reveal any significant QTL affecting this trait (data not shown).

HIF lines, derived from RIL88 (a = S6 and b = S7) and RIL13 (S7), were grown in blue light to confirm QTL for the blue light response on chromosomes 4 and 5. Fifty to 80 seedlings were measured for each homozygous genotype. In addition, the chromosome 4 QTL was confirmed with a random subset of 80 EstC AI-RILs under a lower fluence of blue light (3.0 μE m^−2^ sec^−1^; data not shown).

In total, over 16,000 seedlings were transferred to an acetate sheet covered with a moist paper towel and scanned on a flatbed scanner. Hypocotyl lengths were measured using NIH Image 1.62 (National Institutes of Health).

### Flowering time analysis

For the KendC population, 12 plants per RIL line were planted in a completely randomized design as previously described [42], [46] in both long-days and short days at 23°C. The growth conditions and the methodology of flowering time measurements have been described [42], [36].

### QTL mapping and statistical analyses

The KendC population was genotyped with 181 SNP markers. The EstC population was genotyped with 45 established SSLP markers [21], [47] and 179 SNP markers [32] Genetic linkage maps were determined using Joinmap 3 [48]; the marker orders agreed with the published Col-0 sequence. QTL analysis was carried out with the R-qtl package (http://www.rqtl.org) implemented in R (http://www.r-project.org), via interval mapping using the EM algorithm [49]. LOD thresholds established using one thousand permutations were used to determine the significance of the QTLs. The bQTL package (http://famprevmed.ucsd.edu:16080/faculty/cberry/bqtl/) in R was used to perform a chi-squared test for marker association (segregation distortion). All other statistical analysis was carried out using JMP (http://www.jmp.com). A summary of the identified QTL is given in Table S4.

## Supporting Information

Table S1 Comparisons of publicly available RIL populations with the the EstC and KendC AI-RIL populations.
(0.03 MB XLS)

Table S2 Genotype information and hypocotyl length data for EstC AI-RILs.
(1.79 MB XLS)

Table S3 Genotype information and flowering time data for KendC AI-RILs.
(0.77 MB XLS)

Table S4 Summary of QTL identified.
(0.01 MB XLS)
